# A MaERF110‐MaMYB308 Transcriptional Module Negatively Regulates Lignin‐Mediated Defence Against Fusarium Wilt in Banana

**DOI:** 10.1111/pbi.70528

**Published:** 2026-01-06

**Authors:** Yuqi Li, Yulin Hu, Weijun Xiao, Liu Yan, Junting Feng, Miaomiao Cao, Yanlin Si, Jinhan Lyu, Yankun Zhao, Kai Li, Yongzan Wei, Huigang Hu, Wei Li, Peitao Lü, Wei Wang, Zhenhai Han, Jianghui Xie

**Affiliations:** ^1^ State Key Laboratory of Tropical Crop Breeding, Chinese Academy of Tropical Agricultural Sciences Institute of Tropical Bioscience and Biotechnology & Sanya Research Institute Haikou China; ^2^ Institute for Horticultural Plants China Agricultural University Beijing China; ^3^ Key Laboratory of Tropical Fruit Biology, Ministry of Agriculture, National Field Genebank for Tropical Fruit, South Subtropical Crop Research Institute, Chinese Academy of Tropical Agricultural Sciences Zhanjiang China

## Abstract

Fusarium wilt of banana (FWB), caused by *Fusarium oxysporum* f. sp. *cubense* (*Foc*), threatens global banana production. Lignin reinforces cell walls against pathogens and lodging, yet its regulatory mechanisms in banana remain elusive. Through genome‐wide association study (GWAS) of lignin content across 184 banana accessions, we identified *MaERF110* (encoding an AP2/ERF transcription factor) as a key negative regulator. Overexpression of *MaERF110* in banana and *Arabidopsis* significantly reduced lignin deposition, impaired plant structural integrity and enhanced susceptibility to *Foc* TR4. Integrative RNA‐seq, yeast one‐hybrid and electrophoretic mobility shift assays revealed that MaERF110 directly binds the *MaMYB308* promoter and activates its transcription. *MaMYB308* overexpression similarly suppressed lignin biosynthesis genes and compromised disease resistance. Mechanistically, *MaERF110*‐overexpression plants exhibited disrupted reactive oxygen species (ROS) homeostasis, with elevated H_2_O_2_ and superoxide anion accumulation, reduced antioxidant enzyme activities and increased cell damage upon pathogen infection. We elucidate a MaERF110‐MaMYB308 transcriptional module that represses lignin biosynthesis and disables lignin‐mediated defence against *Foc* TR4. This pathway highlights dual roles for lignin in plant architecture and pathogen defence, providing targets for breeding resistant banana cultivars.

## 1 Introduction

Banana (*Musa* spp.) is a globally critical fruit crop and a staple food for millions of people (FAO 2023). Sustained population growth demands continuous increases in yield, yet production is severely constrained by Fusarium wilt of banana (FWB) and lodging (Bubici et al. 2019; Deng et al. 2021). FWB, caused by *Fusarium oxysporum* f. sp. *cubense* (*Foc*), particularly the devastating tropical race 4 (*Foc* TR4) that infects nearly all cultivars (Bubici et al. 2019). Unfortunately, there is currently no effective chemical or agronomic control. In addition to diseases, lodging is another major constraint in banana production, as the tall banana plants are highly vulnerable to wind damage, leading to yield reduction (Deng et al. 2021). Critically, lignin, a major component of secondary cell walls, provides mechanical support against lodging while serving as a physical barrier against pathogen invasion (Hussain et al. 2019; Moura et al. 2010). Consequently, enhancing lignin biosynthesis has emerged as a dual‐target strategy to combat both challenges (Hua et al. 2025).

The lignin biosynthesis pathway is tightly regulated by transcription factors (TFs), among which AP2/ERF and MYB families play prominent roles. AP2/ERF proteins contain the characteristic AP2 DNA‐binding domain and integrate developmental and environmental cues (Feng et al. 2020). In several species, AP2/ERF TFs have been shown to modulate lignin deposition either positively or negatively. For instance, EjAP2‐1 promotes fruit lignification in *Eriobotrya japonica* via EjMYB interaction (Zeng et al. 2015), *AtRAP2.6* activates wound‐induced lignin synthesis in *Arabidopsis* (Xu et al. 2024), while PagERF81 directly or indirectly inhibits multiple lignin biosynthesis‐related genes in poplar, reducing secondary cell wall lignin deposition and affecting xylem vessel cell density and size (Zhao et al. 2023). Notably, these AP2/ERF regulators often function synergistically with MYB TFs, a family extensively characterised as lignin arbitrators. Specifically, R2R3‐MYB proteins orchestrate the expression of core phenylpropanoid pathway genes. Activator clades such as *Arabidopsis* MYB58/63 up‐regulate nearly the entire lignin pathway, whereas repressor clades typified by MYB4 suppress multiple enzymatic steps (Zhao and Dixon 2011). Beyond development, certain MYB TFs are also implicated in defence‐induced lignification. For example, in rice, OsMYB30 positively regulates lignin accumulation and enhances resistance to insect by binding to the promoters of lignin biosynthetic genes (He et al. 2020). Similarly, in pear, the TF PcMYB44 enhances resistance to fungal pathogens by repressing *PcMIR397* expression and activating the downstream *PcmiR397‐PcLACs* module, which promotes defence‐related lignification (Yang et al. 2023). These findings suggest that MYB TFs serve as central nodes coordinating lignin biosynthesis in both developmental and defence contexts. In banana, *MusaMYB31* negatively regulates lignin biosynthesis (Tak et al. 2017), yet potential ERF‐MYB cooperative modules, especially those linking lignin biosynthesis to disease resistance, remain unexplored.

To identify such regulators in banana, we leveraged the power of genome‐wide association study (GWAS), a proven strategy for dissecting the genetic architecture of complex traits like lignin content (Li et al. 2013; Wen et al. 2014). In crops, GWAS has uncovered causal loci controlling lignin content and composition (Bryant et al. 2023; Lee et al. 2016; Wei et al. 2017). For example, a GWAS conducted in a natural aspen population revealed key genes and regulatory networks—including TFs such as HB5 and VND1—associated with natural variation in lignin traits, elucidating novel genetic mechanisms underlying wood lignification (Luomaranta et al. 2024; Shu et al. 2024). Similarly, in Tartary buckwheat, GWAS pinpointed *FtCOMT1* as a critical gene responsible for enhancing S‐lignin accumulation, which reduces seed hull hardness and offers a promising target for improving dehulling efficiency (Yang et al. 2023). In brown midrib maize, multiple lignin‐related genes, including *β‐glucosidase*, *hct11* and *knox1*, were identified via GWAS as key regulators of Northern leaf blight resistance, providing valuable targets for enhancing pathogen tolerance without compromising silage quality (Kolkman et al. 2023). Furthermore, GWAS applied to *Brassica napus* identified *SHINE1*, an ERF‐family TF, as a major regulator of lignin biosynthesis and lodging resistance, highlighting its potential for improving stem strength in oilseed crops (Wei et al. 2017). Collectively, these studies underscore the power of GWAS in uncovering key transcriptional regulators that coordinately modulate lignin biosynthesis and stress resilience. Despite these significant advances in multiple major crops, parallel comprehensive studies in banana remain scarce, leaving a critical knowledge gap in understanding the genetic basis of lignin‐mediated disease resistance in this globally important fruit crop.

Here, we bridge this gap by measuring lignin content across 184 banana accessions and performed high‐resolution GWAS. We identified *MaERF110* as a major negative regulator of lignin accumulation and demonstrated that it directly activates the repressor MYB TF *MaMYB308*, forming a MaERF110‐MaMYB308 transcriptional module that attenuates lignin biosynthesis and compromises resistance to *Foc* TR4. This study unveils a transcriptional hierarchy that compromises lignin‐mediated barriers against *Foc* TR4, providing a mechanistic basis for breeding resistant bananas.

## 2 Results

### 2.1 GWAS Identifies MaERF110 as a Major Negative Regulator of Lignin Biosynthesis in Bananas

**FIGURE 1:**
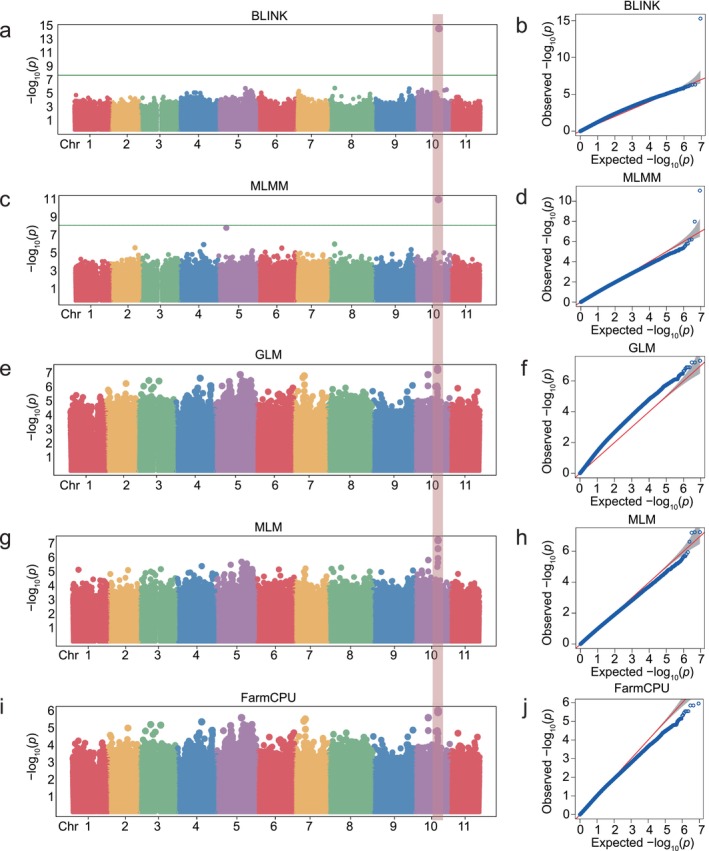
Genome‐wide association mapping of lignin content in banana using five complementary models. (a, c, e, g, i) Manhattan plots generated with BLINK (a), MLMM (c), GLM (e), MLM (g) and FarmCPU (i). The dashed horizontal line shows the Bonferroni‐adjusted significance threshold [−log_10_(*p*) = 8.252]. Pink shading highlights chromosomal regions surpassing this threshold. (b, d, f, h, j) Quantile‐quantile plots for each model showing the distribution of observed versus expected −log_10_(*p*) values.

**FIGURE 2:**
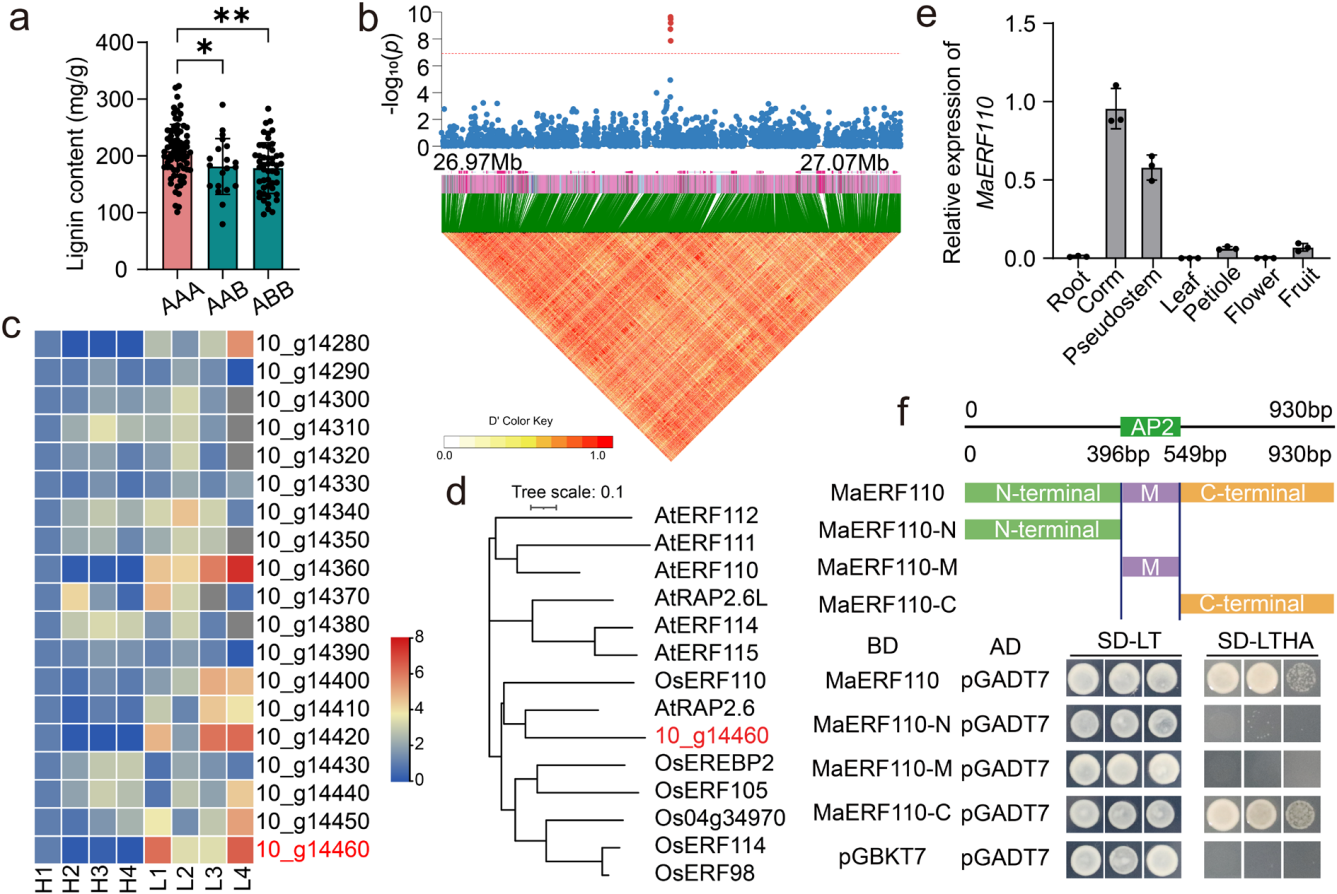
Identification of *MaERF110* as a key regulator associated with lignin content in banana. (a) Lignin content of AAA, AAB and ABB genotypes. (b) Regional Manhattan plot and linkage disequilibrium (LD) heatmap (26.97–27.07 Mb on chromosome 10) from the EMMAX‐MLM model. The dashed horizontal line indicates the significance threshold [−log_10_(*p*) = 6.92], determined by 1000 permutation tests. The significant‐associated SNPs were highlighted in red. LD strength is indicated by the white‐to‐red scale. (c) Expression heatmap of positional candidate genes in four high‐lignin (H1–H4) and four low‐lignin (L1–L4) accessions. Expression values (blue = low, red = high) are normalised to H1. (d) Phylogenetic analysis of *MaERF110* (Macam4_10_g14460) and its homologous genes in *Arabidopsis thaliana* and *Oryza sativa* . (e) Tissue‐specific expression of *MaERF110* relative to *MaActin*. Data are means ± SD (*n* = 3). (f) Transactivation assay of MaERF110 and its truncations in yeast. Growth on SD‐Leu‐Trp (control) and SD‐Leu‐Trp‐His‐Ade (selective) plates after 3 days demonstrates the C‐terminal activation domain. BD, empty pGBKT7 vector.

To dissect the genetic architecture underlying natural variation of lignin accumulation in banana, we quantified lignin content in pseudostem tissues across 184 diverse accessions (Table S1). GWAS employing five models (BLINK, MLMM, GLM, MLM and FarmCPU) consistently detected a significant association signal on chromosome 10 (Figure 1). Notably, AAA‐genotype bananas exhibited significantly higher lignin content than AAB/ABB genotypes, indicating selection for enhanced structural integrity during domestication (Figure 2a).

Within the associated linkage disequilibrium (LD) block (26.97–27.07 Mb), 19 protein‐coding genes were annotated in the *M. acuminata* DH Pahang (v4.3) reference genome (Figure 2b; Table S2). Expression analysis of these candidates in eight accessions spanning the lignin‐content spectrum revealed five genes (*Macma4_10_g14280*, *Macma4_10_g14360*, *Macma4_10_g14400*, *Macma4_10_g14410*, *Macma4_10_g14420* and *Macma4_10_g14460*) whose transcript abundance was inversely correlated with lignin levels (Figure 2c). Among them, *Macma4_10_g14460* encoded a 309‐aa AP2/ERF TF, designated MaERF110. Phylogenetic analysis placed MaERF110 within a clade containing the stress‐responsive *OsERF110* (LOC_Os11g06770) from rice and lignin regulator *AtRAP2.6* (At1g43160) from *Arabidopsis* (Krishnaswamy et al. 2011; Tiwari et al. 2021; Xu et al. 2024; Yongqi et al. 2020) (Figure 2d). Given *AtRAP2.6*'s role in lignin biosynthesis, we hypothesised that *MaERF110* similarly modulates lignin pathways.

To characterise *MaERF110* in the banana, we first analysed its spatiotemporal expression. Quantitative real‐time PCR (qRT‐PCR) revealed ubiquitous expression across tissues, with the highest abundance in corms and pseudostems, key sites for lignin deposition and pathogen defence (Figure 2e). Subcellular localization confirmed MaERF110‐GFP fusion protein localised exclusively to the nucleus (Figure S1). Yeast transactivation assays demonstrated that full‐length MaERF110 drives robust transcriptional activation activity (Figure 2f). Domain dissection revealed this activity resides exclusively in the C‐terminal region (aa 184–309; MaERF110‐C) (Figure 2f). Collectively, these data nominate MaERF110 as a transcriptional activator whose expression in lignifying tissues is negatively associated with lignin deposition.

### 2.2 *MaERF110* Negatively Regulates Lignin Biosynthesis in Banana

**FIGURE 3:**
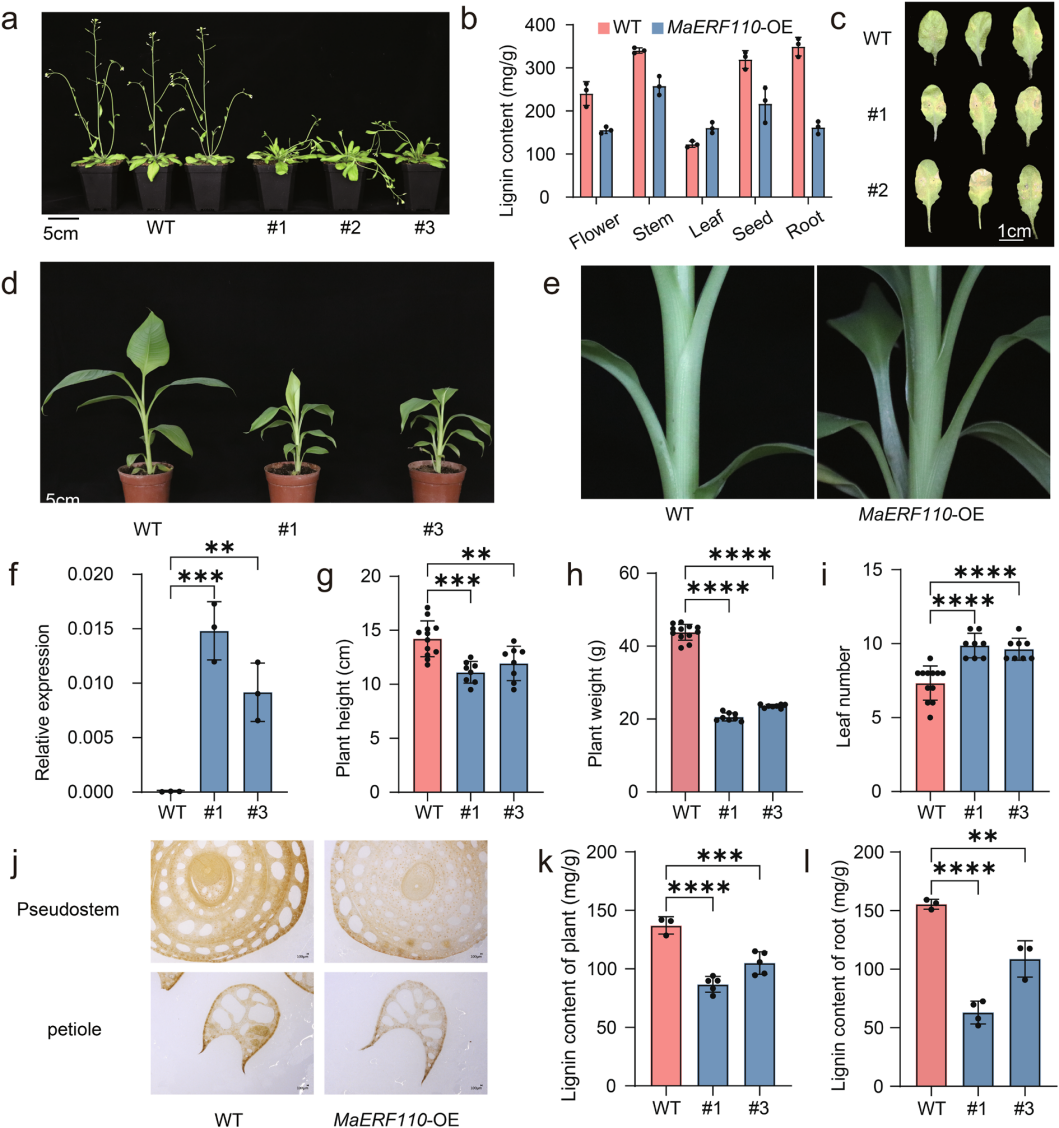
*MaERF110* negatively regulates the lignin content and plant architecture in both *Arabidopsis* and banana. (a) Comparison of plant phenotypes between WT (Col‐0) and *MaERF110*‐OE lines in *Arabidopsis*. Scale bars = 5 cm. (b) Lignin contents in root, stem, leaf, flower and seed of *MaERF110*‐OE *Arabidopsis* lines. Data are means ± SD (*n* = 3). (c) *Foc* TR4 disease symptoms on *MaERF110*‐OE *Arabidopsis* lines and WT at 8 days post‐inoculation (dpi). Scale bar = 1 cm. (d, e) Comparison of plant (d) and pseudostem (e) phenotype between WT and *MaERF110*‐OE lines in banana. Scale bars = 5 cm. (f) *MaERF110* transcript levels in WT and two independent OE lines. Data are means ± SD (*n* = 3). (g, h) plant height (g) and weight (h) of banana. Data are means ± SD (*n* ≥ 8). (i) leaf number per banana plant. Data are means ± SD (*n* ≥ 8). (j) Phloroglucinol‐HCl staining of pseudostem cross‐sections (upper) and petiole sections (lower). Scale bars = 100 μm. (k, l) Quantification of lignin content in *MaERF110*‐OE banana plant (k) and root (l). Data are means ± SD (*n* ≥ 3). Asterisks represent significant differences (*p < 0.05; **p < 0.01; ***p < 0.001; ****p < 0.0001).

To functionally characterise *MaERF110*, we generated *Arabidopsis thaliana* overexpression lines (*35S:MaERF110‐eGFP*, OE) (Figure 3a). Three independent OE lines exhibited 21.64‐ to 28.55‐fold higher *MaERF110* transcript levels than wild‐type (WT) controls (Figure S2a). Phenotypic analysis revealed that *MaERF110*‐OE plants exhibited reduced plant height, increased branching and pronounced stem bending relative to WT (Figure 3a; Figure S2b,c). Histochemical staining with phloroglucinol‐HCl and lignin quantification demonstrated reduced lignin deposition in stems and other tissues of OE lines (Figure 3b; Figure S2d). Consistent with this, expression of key lignin pathway genes (*4CL1*, *C3H*, *C4H*, *CCOMT*, *CCR1*, *cesA*, *COMT*, *F5H*, *PAL1*) was significantly downregulated in *MaERF110*‐OE lines (Figure S2e). Given the role of lignin in pathogen resistance, we challenged plants with *Foc* TR4. *MaERF110*‐OE lines displayed enhanced susceptibility, as evidenced by more severe disease symptoms (Figure 3c). These results indicate that *MaERF110* negatively regulates lignin synthesis and disease resistance in *Arabidopsis thaliana* .

To validate *MaERF110*'s function in its native system, we generated two independent *MaERF110*‐OE lines in the AAB cultivar ‘Xiangfen’ (Figure 3d,e). Transgenic lines exhibited significantly elevated *MaERF110* transcript levels compared to wild‐type plants (Figure 3f). These lines displayed reduced plant height and biomass, along with increased leaf production (Figure 3g–i). Notably, the pseudostems of *MaERF110*‐OE plants showed structural weakness, with leaf sheaths failing to tightly encircle the stem axis (Figure 3e). In addition, histochemical analysis of pseudostem and leaf sheath cross‐sections revealed markedly diminished lignin staining in transgenic lines compared to wild‐type controls (Figure 3j). Quantitative lignin assays confirmed significantly reduced lignin content in plant and root (Figure 3k,l), establishing *MaERF110* as a negative regulator of lignification in banana.

### 2.3 *MaERF110* Disrupts ROS Homeostasis and Enhances Susceptibility to *Foc* TR4

**FIGURE 4:**
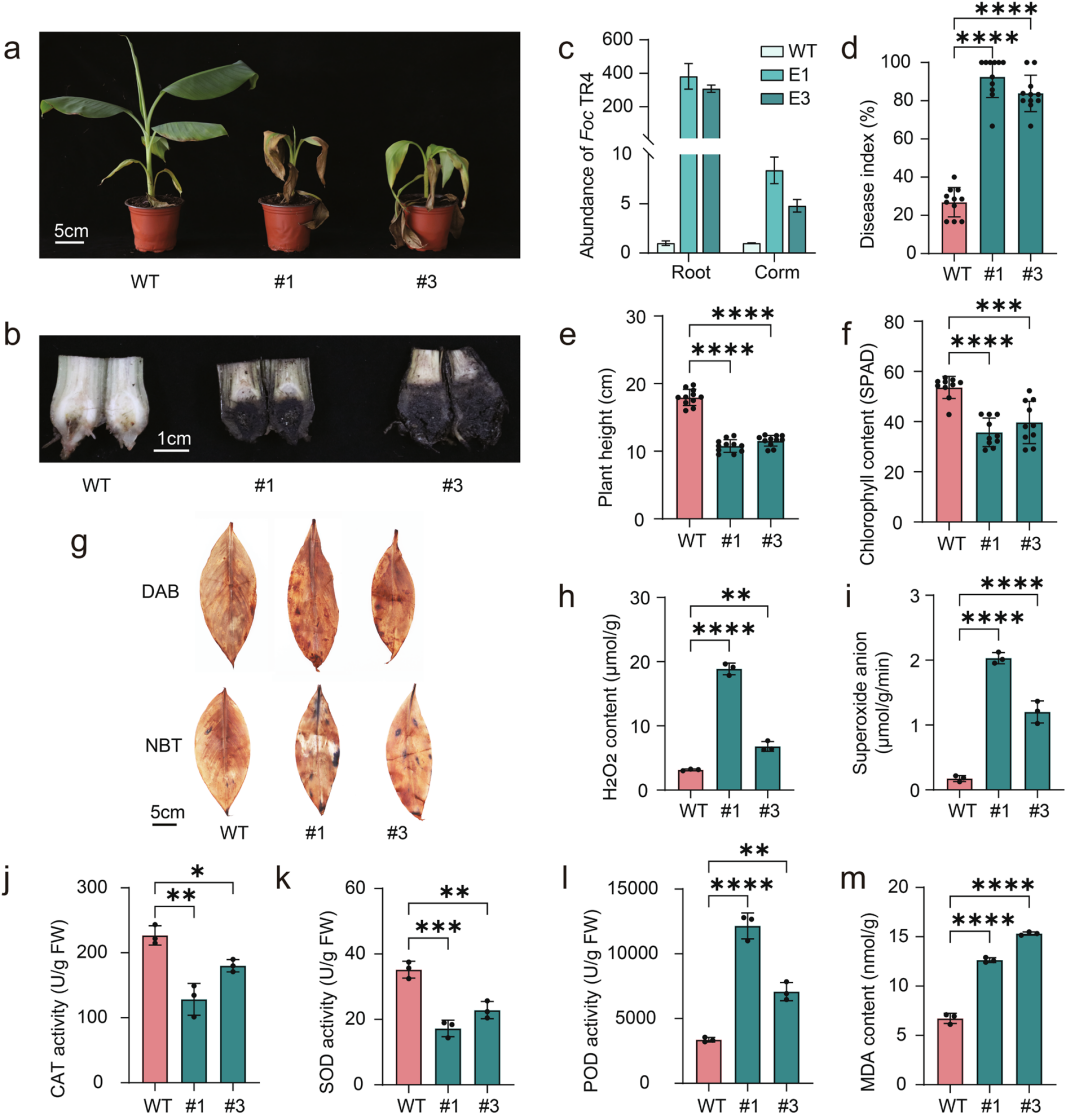
*MaERF110* overexpression compromises banana resistance to *Foc* TR4 and disrupts ROS homeostasis. (a, b) *MaERF110*‐OE banana whole plant (a) and corm (b) phenotypes 15 days after *Foc* TR4 inoculation. Scale bars = 5 cm (a) and 1 cm (b). (c) Quantitative PCR quantification of *Foc* TR4 biomass in roots and corms. (d) Disease index calculated from 11 symptomatic plants per genotype. (e, f) Plant height (e) and chlorophyll content (f) in *MaERF110*‐OE plants after *Foc* TR4 infection (*n* ≥ 10). (g) In situ accumulation of H_2_O_2_ (DAB staining) and superoxide anion (NBT staining) in leaves of *MaERF110*‐OE plants post *Foc* TR4 infection. Scale bars = 5 cm. (h, i) Quantitative levels of H_2_O_2_ (h) and superoxide anion (i) in *MaERF110*‐OE lines after *Foc* TR4 infection. (j–m) Activity of catalase (CAT, j), superoxide dismutase (SOD, k), peroxidase (POD, l) and malondialdehyde (MDA) content (m) in *MaERF110*‐OE lines. Data are means ± SD (*n* = 3). Asterisks represent significant differences (*p < 0.05; **p < 0.01; ***p < 0.001; ****p < 0.0001).

To determine how lignin deficiency translates into compromised disease resistance, we challenged WT and *MaERF110*‐OE banana plants with *Foc* TR4. By 15 days post‐inoculation (dpi), OE lines displayed severe leaf wilting (Figure 4a), pronounced browning of roots and corms (Figure 4b), and > 300‐fold greater fungal biomass in roots and > 4‐fold in corms relative to WT (Figure 4c). Disease indices corroborated these visual symptoms (Figure 4d). Infected OE plants also showed exacerbated growth suppression and chlorophyll loss compared to WT controls (Figure 4e,f), indicating that reduced lignin and impaired pathogen defence act synergistically to compromise plant performance.

We next examined whether the heightened susceptibility was associated with perturbed ROS signalling. DAB and NBT histochemical staining revealed excessive accumulation of hydrogen peroxide (H_2_O_2_) and superoxide anions (^·^O_2_^−^) in *MaERF110*‐OE leaves at 15 dpi (Figure 4g), indicating impaired ROS scavenging. Quantitative assays confirmed 2–6‐fold increases in both ROS species in transgenic lines (Figure 4h,i). Concomitant with this oxidative burst, catalase (CAT) and superoxide dismutase (SOD) activities were 20%–43% lower in OE lines, whereas peroxidase (POD) activity was significantly elevated, indicating a compensatory but ineffective scavenging response (Figure 4j–l). This antioxidant imbalance correlated with severe oxidative damage, evidenced by increased malondialdehyde (MDA) accumulation, which increased by 1.88‐fold and 2.28‐fold compared with that in WT plants (Figure 4m). Collectively, these results demonstrate that *MaERF110‐*mediated lignin suppression weakens the physical barrier to *Foc* TR4 and simultaneously deregulates ROS homeostasis, culminating in accelerated cell death and heightened pathogen susceptibility.

### 2.4 MaERF110 Directly Activates *MaMYB308* via a GCC‐Box in Its Promoter

**FIGURE 5:**
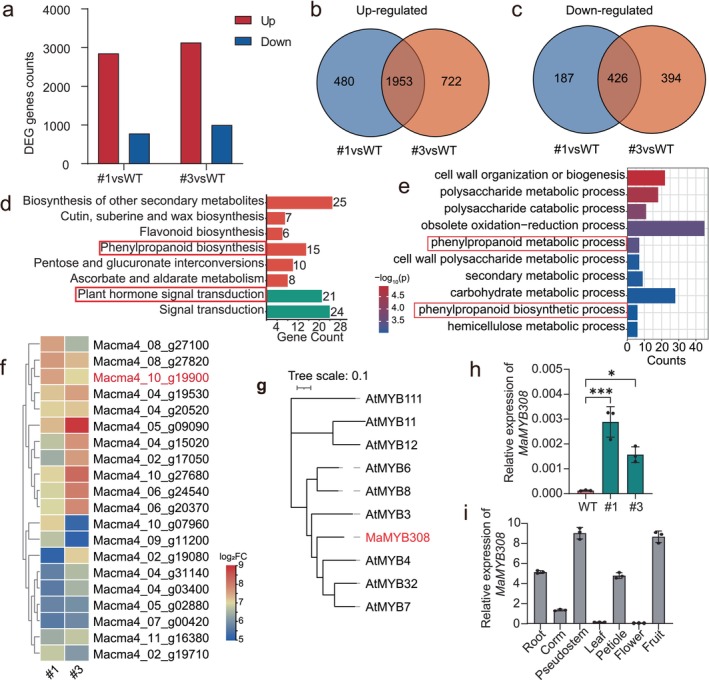
Genome‐wide identification: *MaMYB308* is a direct target of MaERF110 through RNA‐seq. (a) Bar plots revealing the DEGs of *MaERF110*‐OE#1 and *MaERF110*‐OE#3 lines compared to WT by RNA‐seq. Up, up‐regulated; Down, down‐regulated. Log_2_FC ≥ |2| and FDR < 0.05 were considered as DEGs (b, c) Venn diagrams showing the overlapping up‐regulated (b) and down‐regulated (c) genes in *MaERF110*‐OE#1 and *MaERF110*‐OE#3 lines. (d, e) Top enriched GO (d) and KEGG (e) terms of MaERF110‐regulated genes as defined by RNA‐seq analysis. (f) Heatmap showing the expression of direct MaERF110‐regulated target genes. (g) Phylogenetic analysis of *MaMYB308* (Macam4_10_g19900) and its homologous genes in *Arabidopsis thaliana* . (h) Expression of *MaMYB308* in *MaERF110*‐OE lines. Data are means ± SD (*n* = 3). Asterisks indicate statistically significant differences (*p < 0.05; **p < 0.01; ***p < 0.001; ****p < 0.0001). (i) Tissue‐specific *MaMYB308* expression relative to *MaActin*. Data are means ± SD (*n* = 3).

To dissect the transcriptional circuitry underlying MaERF110‐mediated lignin suppression, we performed RNA sequencing (RNA‐seq) on pseudostems of *MaERF110*‐OE and WT bananas. Comparative RNA‐seq of two independent OE lines revealed 1953 consistently up‐regulated and 426 down‐regulated genes (Figure 5a–c). Gene ontology (GO) and KEGG analyses highlighted enrichment in phenylpropanoid biosynthesis and cell wall organisation (Figure 5d,e), implicating *MaERF110* in lignification pathways. Among 2379 differentially expressed genes (DEGs), the R2R3‐MYB repressor *MaMYB308* (Macma4_10_g19900) exhibited strongly upregulated expression (Figure 5f). Phylogenetic analysis confirmed *MaMYB308* clusters with *Arabidopsis* homologues (*AtMYB3/4/7*/*32*) (Figure 5g), which globally inhibit lignin biosynthesis. qRT‐PCR analysis confirmed that *MaMYB308* expression was significantly elevated in *MaERF110*‐OE lines, consistent with the RNA‐seq data (Figure 5h). Tissue‐specific qRT‐PCR showed *MaMYB308* is highly expressed in pseudostems and corms (Figure 5i), mirroring *MaERF110*'s expression pattern.

**FIGURE 6:**
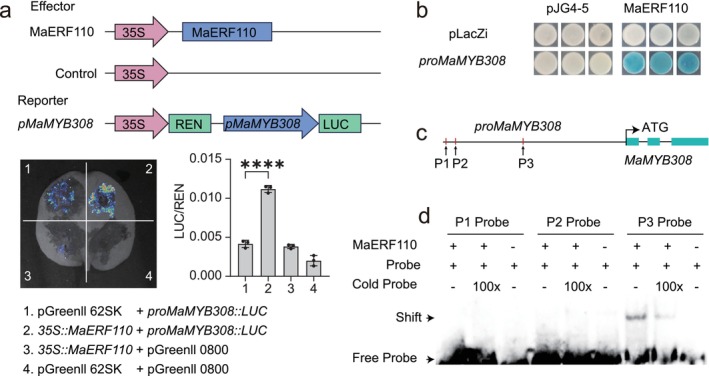
MaERF110 enhances the expression of *MaMYB308* in banana. (a) Dual‐luciferase assay demonstrating that MaERF110 directly activates the promoter of *MaMYB308*. LUC/REN ratios are means ± SD (*n* = 3). (b) Yeast one‐hybrid assay showing MaERF110 binding to the *MaMYB308* promoter. Three independent yeast colonies per treatment were photographed at 3 days post‐incubation (dpi). (c) Schematic diagram of the *MaMYB308* promoter region, highlighting the putative MaERF110 binding site (red lines). (d) Electrophoretic mobility shift assay (EMSA) confirming specific binding of GST‐MaERF110 to the biotin‐labelled *MaMYB308* probe.

We next validated the regulatory relationship between MaERF110 and *MaMYB308*. Dual‐luciferase assays in *Nicotiana benthamiana* leaves demonstrated that MaERF110 elevated *MaMYB308* promoter activity (Figure 6a). Yeast one‐hybrid (Y1H) assays confirmed this interaction (Figure 6b). Promoter analysis identified three GCC‐like *cis*‐elements; electrophoretic mobility shift assay (EMSA) revealed specific binding of MaERF110 to the P3 probe (Figure 6c,d). These results showed that MaERF110 directly binds to the *MaMYB308* promoter through the GCC‐like box to activate its transcription, establishing a MaERF110‐MaMYB308 regulatory module that represses lignin biosynthesis.

### 2.5 *MaMYB308* Is a Conserved Negative Regulator of Lignin Biosynthesis and Disease Resistance

**FIGURE 7:**
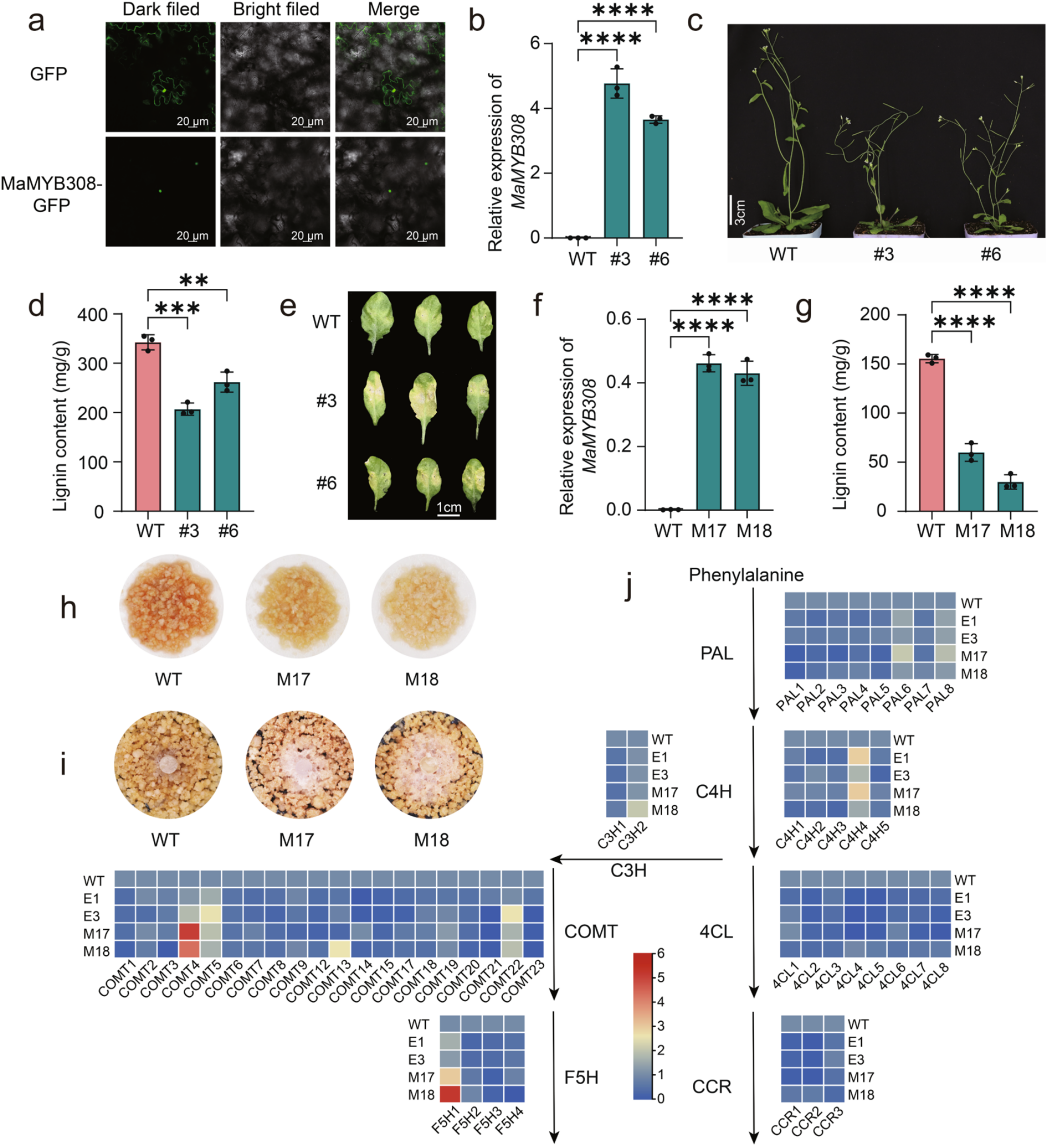
*MaMYB308* functions as a negative regulator of lignin biosynthesis and *Foc* TR4 resistance. (a) Nuclear localization of MaMYB308‐GFP in *Nicotiana benthamiana* leaves epidermal cells. Scale bars = 20 μm. (b) qRT‐PCR confirmation *MaMYB308* overexpression in *Arabidopsis*. Data are means ± SD (*n* = 3). (c) Phenotypic comparison between WT and *MaMYB308*‐OE lines in *Arabidopsis*. Scale bars = 5 cm. (d) Total lignin content in *MaMYB308*‐OE *Arabidopsis* stems. Data are means ± SD (*n* = 3). (e) Disease symptoms in *MaMYB308*‐OE lines at 8 days post‐inoculation (dpi) with *Foc* TR4. Scale bar = 1 cm. (f) *MaMYB308* transcript abundance in transgenic banana callus lines. Data are means ± SD (*n* = 3). (g) Lignin content in *MaMYB308*‐OE calli. Data are means ± SD (*n* = 3). (h) Phloroglucinol–HCl staining of callus sections showing reduced lignin deposition. (i) *Foc* TR4 necrosis on banana callus 10 dpi. (j) Expression of core lignin biosynthesis genes (*PAL*, *C4H*, *4CL*, *CCR*, *C3H*, *COMT*, *F5H*) in *MaERF110*‐ and *MaMYB308*‐OE banana lines relative to WT. Data are means ± SD (*n* = 3). Asterisks denote statistically significant differences (*p < 0.05; *p < 0.01; **p < 0.001; ***p < 0.0001).

Subcellular localization confirmed nuclear localization of MaMYB308‐GFP in *N. benthamiana* (Figure 7a), consistent with its predicted function as a TF. Given the established role of its *Arabidopsis* homologue *AtMYB4* in suppressing lignin biosynthesis (Xiao et al. 2021), we generated *Arabidopsis* lines constitutively expressing *MaMYB308* under the CaMV 35S promoter. Two independent OE lines (#3 and #6) exhibited 248.68 to 266.77‐fold higher *MaMYB308* transcripts than WT controls (Figure 7b). Recapitulating the *MaERF110*‐OE phenotype, these plants displayed pronounced stem bending (Figure 7c), and a 61.52%–80.76% reduction in total lignin (Figure 7d). When challenged with *Foc* TR4, detached leaves of *MaMYB308*‐OE lines developed necrotic lesions that were significantly larger than those on WT leaves (Figure 7e), demonstrating that *MaMYB308* overexpression compromises both lignification and disease resistance in Arabidopsis.

To further validate *MaMYB308* function in banana, we produced two independent transgenic callus lines (M17 and M18) overexpressing *MaMYB308* (Figure 7f). Phloroglucinol‐HCl staining revealed a marked reduction in lignin autofluorescence and a significant decrease in lignin content relative to WT callus (Figure 7g,h). Pathogen inoculation assays showed that *Foc* TR4 colonisation was significantly more extensive on *MaMYB308*‐OE callus than on WT controls (Figure 7i).

We next examined whether *MaMYB308*, like its *Arabidopsis* homologue *AtMYB4*, represses the core lignin synthesis pathway. qRT‐PCR analysis revealed that transcript levels of *PAL, C4H, 4CL, CCR, C3H, COMT* and *F5H* were down‐regulated to comparable extents in both *MaERF110*‐ and *MaMYB308*‐overexpressing lines in banana (Figure 7j). Collectively, these data establish *MaMYB308* as a transcriptional brake on lignin biosynthesis and pathogen defence, operating downstream of MaERF110 in the banana lignin regulatory network.

## 3 Discussion

Lignin is a double‐edged polymer; it fortifies cell walls against mechanical stress and pathogen ingress, yet its excessive accumulation can compromise biomass digestibility. Deciphering how plants fine‐tune lignin deposition is therefore pivotal for sustainable agriculture. Here, we provide a genome‐to‐phenome dissection of lignin regulation in banana, a crop whose global production is increasingly threatened by Fusarium wilt. By integrating GWAS, multi‐omics and functional genetics, we uncover a transcriptional module, MaERF110‐MaMYB308, that operates as a tunable brake on lignin biosynthesis and, consequently, on disease resistance.

Although GWAS has successfully dissected loci related to traits including plant architecture, stress response, yield and metabolism in crops such as maize (Wang et al. 2023), rice (Yu et al. 2023) and rapeseed (Hu et al. 2022), achieving significant progress. However, GWAS‐based research on lignin‐related genes mainly focuses on cereals and woody perennials, such as rice (Bao et al. 2007; Panahabadi et al. 2022), maize (Kolkman et al. 2023), poplar (Bryant et al. 2023; Luomaranta et al. 2024), rapeseed (Wei et al. 2017) and Tartary buckwheat (Yang et al. 2024), its application to lignin regulation in banana has been limited. Our study fills this critical gap by applying GWAS to 184 banana accessions, directly linking natural variation in pseudostem lignin content to *MaERF110* (Figure 1). This approach moves beyond candidate gene studies and positions *MaERF110* as a major negative regulator identified through an unbiased genome‐wide screen in *Musa*. Functional validation in both banana and *Arabidopsis* confirmed its conserved role in repressing lignification (Figures 3 and 7; Figure S2e). Intriguingly, lignin content showed significant genotype dependence, with AAA‐genotype samples exhibiting markedly higher levels than AAB and ABB genotypes (Figure 2a), suggesting that selection for structural traits during the domestication of dessert bananas may have inadvertently shaped lignin‐associated alleles. This provides a genetic basis and a potential breeding target for manipulating this trait.

The core novelty of our work lies in the elucidation of a previously uncharacterized transcriptional hierarchy in banana, where an AP2/ERF TF directly governs a repressor MYB to coordinately suppress lignin‐mediated defence. While *MusaMYB31* was previously reported as a negative regulator of lignin (Tak et al. 2017), and *MaMYB308* homologues are known repressors in other species (Zhao and Dixon 2011), the upstream regulator orchestrating their expression in the context of pathogen defence in banana remained unknown. Here, we demonstrate that MaERF110 is not merely a parallel regulator but sits upstream, directly activating *MaMYB308* transcription by binding to its promoter (Figure 6). This establishes a clear ERF‐to‐MYB repressor cascade that amplifies the suppression signal on the lignin biosynthetic pathway. This hierarchical module is distinct from the known role of *MusaMYB31* and provides a more integrated regulatory layer (Tak et al. 2017), explaining how developmental or environmental signals perceived by AP2/ERF TFs can be transduced into precise transcriptional repression of lignin synthesis and compromised disease resistance in banana.

As a major structural component of cell walls, lignin serves as a physical barrier against pathogen infection (Moura et al. 2010). Substantial evidence indicates that elevated lignin levels enhance resistance to multiple pathogens (Gallego‐Giraldo et al. 2011; Zhou et al. 2024), while reduced lignin content exacerbates pathogen susceptibility (Xiao et al. 2024). Fusarium wilt caused by *Fusarium oxysporum* f. sp. *cubense* poses the most severe threat to global banana production (Dean et al. 2012). Comparative transcriptomics confirmed enrichment of lignin and flavonoid biosynthesis pathways in the Cavendish cultivar ‘Brazilian’ following *Foc*1 infection (Dong et al. 2020). The resistant cultivar ‘Goldfinger’ triggers defence mechanisms through enhanced root lignin deposition upon *Foc* TR4 challenge (De Ascensao and Dubery 2000), while in resistant cultivar ‘Jin Long’, *Foc* TR4 infection activates lignin biosynthesis genes *CCoAOMT* and *COMT* (Duan et al. 2025). These studies collectively demonstrate the positive regulatory role of lignin in banana pathogen resistance. Our pathogen inoculation experiments further showed that *MaERF10*‐OE lines with reduced lignin content, increased H_2_O_2_ and ^·^O_2_^−^ accumulation and suppressing antioxidant enzyme activity (Figure 4).

AP2/ERF TFs are well‐known integrators of hormone signalling, particularly ethylene (ET) and jasmonic acid (JA) (Ma et al. 2020). The placement of MaERF110 within this regulatory network opens new avenues for understanding the hormonal control of lignification and defence in banana. It is plausible that MaERF110 itself is induced by ET or JA signals upon *Foc* TR4 perception. This would position the MaERF110‐MaMYB308 module as a potential negative feedback loop within defence hormone networks. Future research should investigate the hormonal inducibility of *MaERF110* and whether this module interacts with salicylic acid (SA) pathways, potentially representing a point of crosstalk where SA‐mediated biotrophic defence is compromised by ET/JA‐responsive lignin repression. This hormonal context adds a dynamic layer to our model, suggesting that the MaERF110‐MaMYB308 module is a key node where development, hormone signalling and pathogen defence converge in banana.

Recent studies have confirmed the regulatory role of AP2/ERF TFs in lignin biosynthesis across multiple species. However, research on AP2/ERF TFs in banana has primarily focused on fruit ripening and responses to biotic/abiotic stresses (Fan et al. 2016; Huang et al. 2024; Teoh et al. 2024; Xiao et al. 2013), with no prior reports linking these TFs to lignin synthesis. Here, we report the first identification of an AP2/ERF TF, *MaERF110*, as a negative regulator of lignin biosynthesis in banana (Figure 3). Intriguingly, its *Arabidopsis* homologue, *AtRAP2.6*, was shown to promote lignin synthesis during wound healing (Xu et al. 2024). This functional antagonism suggests that the wiring of AP2/ERF‐lignin circuits may have diverged between monocots and dicots, possibly reflecting distinct cell‐wall architectures or pathogen pressures. Our finding in banana, a monocot, highlights the species‐specific nature of these regulatory networks and cautions against extrapolating functions from model dicots.

The pathways governing lignin biosynthesis and metabolism have been extensively studied, with the functions of associated genes well characterised. Within the lignin regulatory network, while multiple TFs contribute to its regulation, the NAC‐MYB regulatory module is widely recognised as the cornerstone of lignin biosynthesis. Among these, MYB TFs have been most extensively investigated, functioning as either activators or repressors to modulate lignin synthesis (Ma and Constabel 2019). In this study, RNA‐seq analyses identified *MaMYB308* as a downstream TF regulated by MaERF110 (Figure 5). Experimental validation revealed that MaERF110, acting as a transcriptional activator, binds to the GCC‐like box in the *MaMYB308* promoter to activate its expression (Figure 6). In *Arabidopsis*, the homologue of *MaMYB308*, *AtMYB4*, plays a critical role in lignin regulation by suppressing the majority of enzymes in the lignin biosynthesis pathway, thereby negatively regulating lignin synthesis. Consistently, *MaMYB308* exhibited similar repressive activity on lignin synthesis in both *Arabidopsis* and banana calli (Figure 7d,g). Fungal inoculation assays further demonstrated that, analogous to *MaERF110*, overexpression of *MaMYB308* enhanced fungal susceptibility in *Arabidopsis* and banana calli (Figure 7i). qRT‐PCR analysis confirmed that overexpression of either *MaERF110* or *MaMYB308* significantly downregulated key enzymes in the lignin biosynthesis pathway (Figure 7j), indicating that *MaMYB308* negatively regulates lignin synthesis by suppressing critical pathway enzymes. These findings collectively demonstrate that MaERF110 modulates lignin content and pathogen resistance by regulating *MaMYB308*, which in turn controls the expression of key lignin biosynthesis enzymes.

**FIGURE 8:**
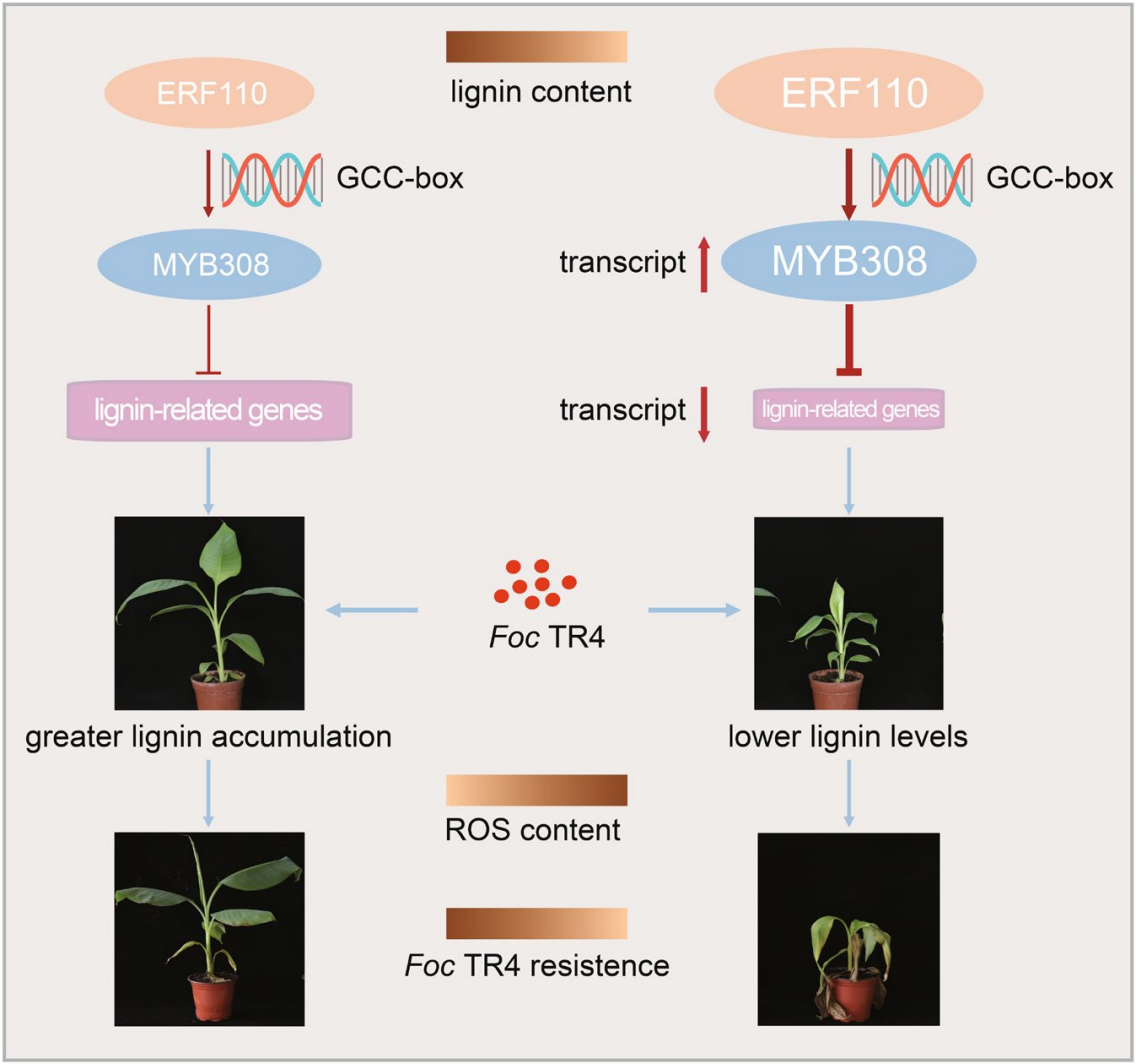
The MaERF110‐MaMYB308 module negatively tunes lignin‐mediated defence against Fusarium wilt in banana. MaERF110 functions as a transcriptional activator that binds a GCC‐box element in the *MaMYB308* promoter, up‐regulating *MaMYB308* expression. MaMYB308 in turn represses the lignin biosynthetic pathway (*PAL*, *C4H*, *4CL*, *CCR*, *C3H*, *F5H*, *COMT*), thereby decreasing lignin deposition in banana cell walls. Reduced lignin compromises structural integrity, disrupts ROS homeostasis and diminishes resistance to *Foc* TR4. Thus, the MaERF110‐MaMYB308‐lignin biosynthetic enzyme module constitutes a key negative regulatory axis modulating both lignin content and *Foc* TR4 resistance in banana.

In summary, we identified the key gene *MaERF110* associated with lignin content in banana through GWAS and proposed a MaERF110‐MaMYB308‐lignin biosynthesis enzymes module that regulates lignin synthesis and disease resistance in banana (Figure 8). This module defines a novel hierarchical relationship in banana where an ERF directly controls a MYB repressor to gatekeep lignin‐mediated defence. MaERF110 directly activates the transcription of *MaMYB308*, which subsequently suppresses the expression of key lignin biosynthesis enzymes, thereby inhibiting lignin synthesis and resistance to *Foc* TR4. The potential embedding of this module within hormone signalling networks further enhances its significance as a regulatory hub. Consequently, the MaERF110‐MaMYB308‐lignin biosynthesis enzymes module demonstrates significant application potential in modulating banana lignin content and *Foc* TR4 resistance. Leveraging the disease resistance conferred by this module, targeted banana breeding strategies can be developed to enhance resistance against *Foc* TR4, thereby safeguarding banana production from Fusarium wilt in future agricultural practices.

## 4 Materials and Methods

### 4.1 Plant Materials and Growth Conditions

A collection of 184 banana (*Musa* spp.) accessions was field‐grown in Danzhou, Hainan, China. For all subsequent transformation and phenotyping experiments, the AAB cultivar ‘Xiangfen’ was used. Shoot cultures were initiated and maintained on Murashige and Skoog (MS) medium supplemented with 0.2 mg/L naphthaleneacetic acid (NAA) and 1.0 mg/L 6‐benzylaminopurine (6‐BA). Cultures were incubated at 24°C ± 2°C under a 16 h light/8 h dark photoperiod and sub‐cultured every 25 days. Embryogenic callus was induced and propagated on hormone‐free MS medium in darkness at 24°C ± 2°C with sub‐culturing every 20 days.

Wild‐type (col‐0) and transgenic lines of *Arabidopsis thaliana* were germinated in petri dishes containing 1/2MS medium. Before sowing, all seeds underwent a treatment with 15% sodium hypochlorite for 12 min, disinfected in 70% ethanol for 30 s and rinsed three times with ddH_2_O. Before sowing, the seeds were vernalized at 4°C in darkness for 3 days, and then placed in a light incubator at 24°C and cultured under a 16 h light/8 h dark cycle.

### 4.2 Plant Transformation and Transgenic Line Identification

The full‐length coding sequence (CDS) of MaERF110 or MaMYB308 (without stop codon) was amplified by high‐fidelity PCR and inserted into pCAMBIA1302 under the control of the CaMV 35S promoter, in‐frame with the C‐terminal GFP tag. The resulting binary vectors were transferred into *Agrobacterium tumefaciens* strain EHA105 by electroporation. Agrobacterium‐mediated transformation of embryogenic cell suspensions of ‘Xiangfen’ was performed as described by Liu et al. (2017). For *Arabidopsis* (Col‐0), floral dip was carried out according to Zhang et al. (2006). Transgenic lines were identified by PCR using gene‐specific primers (Table S3) and propagated for molecular and phenotypic analysis.

### 4.3 Lignin Quantification

The 184 banana accessions for the GWAS were field‐grown under standard agricultural conditions. Pseudostem tissues were sampled at the vegetative growth stage. For the transgenic validation studies, plants were grown in pots under greenhouse conditions to ensure environmental control. Pseudostem tissues were also collected at a comparable vegetative growth stage for consistency. To measure the lignin content of the GWAS population and transgenic plants, the samples were first dried at 80°C until achieving a constant weight. After drying, the samples were finely ground and sieved through a 40‐mesh sieve. Subsequently, precisely 3 mg of the sieved material was collected for lignin content analysis. Total lignin was determined spectrophotometrically using the Lignin Content Test Kit (Solarbio, China). This kit is based on the acetyl bromide method, and the assays were performed strictly according to the manufacturer's instructions. To ensure result accuracy, each sample was subjected to three replicates.

For lignin histochemical staining, fresh tissue from banana pseudostems, leaf sheaths and *Arabidopsis* stems was used. The free‐hand cross‐sections of tissues were used for staining following the lignin staining (Phloroglucinol‐HCl) kit (Yuanye Bio‐Technology, China), and images were captured by microscope.

### 4.4 Genome‐Wide Association Study (GWAS)

Genomic DNA from 184 banana accessions was re‐sequenced, yielding 8.9 million high‐quality SNPs (minor allele frequency ≥ 0.05). Association analyses were performed with the R package GAPIT (Wang and Zhang 2021) using five complementary models: BLINK, MLMM, GLM, MLM and FarmCPU, and independently corroborated with the efficient mixed‐model association expedited (EMMAX) software (Kang et al. 2010). Significant SNPs were aggregated into linkage‐disequilibrium (LD) block with LDblockshow (Dong et al. 2021). Candidate genes within each LD interval were functionally annotated and homologous in *Arabidopsis* and rice identified by TBtools (Chen et al. 2020).

### 4.5 Phylogenetic Analysis

The protein sequences of 14 AP2/ERF TFs from banana ( *Musa acuminata* ), *Arabidopsis thaliana* and *Oryza sativa* were obtained from the following public databases: Banana Genome Hub (https://banana‐genome‐hub.southgreen.fr/), The *Arabidopsis* Information Resource (TAIR; https://www.arabidopsis.org/) and the National Center for Biotechnology Information (NCBI; https://www.ncbi.nlm.nih.gov/). A phylogenetic tree was constructed using ClustalW and the neighbour‐joining method in MEGA7.0 software, with bootstrap support based on 1000 replicates (Data S1 and S2). A phylogenetic tree of 10 MYB TFs was constructed using the same method (Data S3 and S4).

### 4.6 Pathogen Inoculation and Disease Evaluation

#### 4.6.1 Inoculum Preparation

The *Foc* TR4 strain was cultured in potato‐dextrose broth (PDB: 200 g potato extract, 20 g glucose, 1 L H_2_O) at 28°C with shaking at 200 rpm for 5 days to generate the inoculum. The resulting spore suspension was quantified using a haemocytometer, and the concentration was adjusted to 1.0 × 10^6^ CFU/mL for all subsequent inoculation assays.

#### 4.6.2 Banana Plant Inoculation and Experimental Design

Two‐ to three‐month‐old plants (with 6–7 leaves) of the WT and two independent transgenic lines (#1 and #3) were used. The root‐wounding method was employed for inoculation with the *Foc* TR4 spore suspension. A completely randomised design was implemented in the greenhouse, with pots containing plants of different genotypes arranged randomly. Each genotype included at least 10 biological replicates (individual plants), and pots were spaced 15 cm apart to ensure adequate air circulation. Following inoculation, plants were maintained in a controlled greenhouse at 28°C with 70% relative humidity and a 14‐h light/10‐h dark photoperiod. Disease severity was assessed at 15 dpi. To prevent assessment bias, disease scoring (including disease index, plant height and chlorophyll content measurements) was performed by an investigator who was blinded to the plant genotypes where feasible.

#### 4.6.3 Growth Conditions

Inoculated plants were maintained in a controlled greenhouse at 28°C with 70% relative humidity and a 14‐h light/10‐h dark photoperiod. Pots were spaced 15 cm apart to ensure adequate air circulation and uniform growth conditions.

#### 4.6.4 Banana Callus Inoculation

Sterile mycelial plugs from actively growing *Foc* TR4 cultures were placed directly onto the surface of embryogenic banana calli. The inoculated calli were incubated at 28°C in darkness, and the diameter of the necrotic area was measured at 10 dpi.

#### 4.6.5 *Arabidopsis* Inoculation

Four‐week‐old soil‐grown plants were inoculated via syringe infiltration on the abaxial side of fully expanded leaves with the *Foc* TR4 spore suspension. Inoculated plants were maintained at 28°C, and the lesion area on detached leaves was scored at 8 dpi.

For all assays, each inoculation experiment was set up with at least three technical replicates, and biological replicate experiments were performed three times independently.

### 4.7 Measurement of ROS Levels and Antioxidant Enzyme Activities

At 15 dpi with *Foc* TR4, the third fully expanded leaf from the top of plants was harvested. For histochemical staining, leaves were immediately used for DAB and NBT staining to detect H_2_O_2_ and superoxide anion (^·^O_2_^−^) accumulation, respectively. For NBT staining, fresh leaves were vacuum‐infiltrated with 0.1 mg/mL NBT (Sangon, China) in 25 mM HEPES buffer (pH 7.8) and incubated at 25°C in darkness for 2 h. For DAB staining, leaves were vacuum‐infiltrated with 0.1 mg/mL DAB (Sangon, China) in 50 mM Tris‐acetate buffer (pH 3.8) and subsequently incubated in darkness for 24 h. Following staining, all leaves were decolorized in 80% ethanol at 70°C until complete chlorophyll removal. Each experiment was independently repeated three times. The quantitative assays for H_2_O_2_, ^·^O_2_^−^, CAT, SOD, POD and MDA were performed using commercial assay kits (Solarbio, China) following the manufacturer's protocols. All quantitative measurements included three independent biological replicates.

### 4.8 Quantitative Real‐Time PCR

Total RNA was extracted from plant samples using the RNAprep Pure Kit (TIANGEN, China), the first‐strand complementary DNA (cDNA) was synthesised with the PrimeScript RT Reagent Kit with gDNA Eraser (Takara, Japan). Quantitative real‐time PCR (qRT‐PCR) was performed on the Roche LightCycler96 PCR instrument (Roche, Switzerland) using the SYBR Premix Ex Taq reagent (Takara, Japan). Banana actin (*Macma4_09_g08610*) and *Arabidopsis* actin (*AT3G18780*) served as endogenous references. Relative expression was calculated using the 2^−ΔΔCt^ method (Livak and Schmittgen 2001). All reactions were performed in three biological replicates. Primer sequences are listed in Table S3.

### 4.9 Subcellular Localization

The *MaERF110*‐eGFP fusion construct was transformed into *Agrobacterium* strain GV3101 and cultured overnight at 28°C in LB medium supplemented with 50 mg/L rifampicin and 50 mg/L kanamycin. Cells were pelleted at 4000 g for 5 min and resuspended in infiltration buffer (10 mM MgCl_2_; 10 mM MES‐KOH, pH 5.5; 100 μM acetosyringone) to an OD_600_ of 0.8. The suspension was pressure‐infiltrated into fully expanded leaves of 4‐week‐old *Nicotiana benthamiana* plants. After 24 h darkness, plants were transferred to standard growth conditions (16 h light/8 h dark) for an additional 48 h. GFP fluorescence was visualised using Inverted Confocal Fluorescence Microscope FV3000 (Olympus, Japan).

### 4.10 Yeast One‐Hybrid (Y1H) Assay

The full‐length *MaERF110* CDS was cloned into the pJG4‐5 prey vector (carrying the GAL4 activation domain), and a *MaMYB308* promoter fragment containing the predicted MaERF110 binding site was inserted into the pLacZi vector upstream of the LacZ reporter. Both constructs were co‐transformed into the yeast strain EGY48 and cultured on SD/‐Trp/‐Ura medium at 28°C for 3 days. Three single colonies per combination were subjected to a 5‐bromo‐4‐chloro‐3‐indolyl‐β‐D‐galactopyranoside (X‐gal) overlay assay. Blue coloration after 4–6 h at 30°C was scored as evidence of protein–DNA interaction. The primers used are listed in Table S3.

### 4.11 Electrophoretic Mobility Shift Assay (EMSA)

*MaERF110* CDS was introduced into pGEX6P‐1 vector to generate an N‐terminal GST fusion. The construct was transformed into *Escherichia coli* BL21 (DE3) and grown in LB medium containing 100 mg/L ampicillin at 37°C to OD_600_ = 0.6–0.8. Expression of GST‐MaERF110 was induced with 0.2 mM isopropyl‐β‐D‐thiogalactoside (IPTG) at 16°C for 8 h. Recombinant protein was purified using BeyoGold GST‐tag Purification Resin Kit (Beyotime, China) according to the manufacturer's instructions. The probes for EMSA assay were synthesised by Sangon Biotech (Shanghai, China) and purified by high‐performance liquid chromatography. The hot probes were 5′‐end‐labelled with biotin, while the cold probes were unlabelled. Mutant probes contained adenine substitutions within the GCC‐box motif. The EMSA assay was carried out using the Light Shift Chemiluminescent EMSA Kit (Beyotime, China) according to the manufacturer's instructions. Probe sequences are listed in Table S3.

### 4.12 Dual‐Luciferase Reporter Assays

The *MaERF110* CDS was cloned into the pGreenII 62‐SK vector (effector), while the *MaMYB308* promoter fragment containing the *MaERF110*‐binding site was inserted into the pGreenII 0800‐LUC vector (reporter). Each construct was transformed into *Agrobacterium tumefaciens* GV3101 (p19) and cultured overnight at 28°C. Cells were pelleted at 4000 g for 10 min and resuspended in infiltration buffer (10 mM MgCl_2_, 10 mM MES‐KOH, pH 5.5; 100 μM acetosyringone) to OD_600_ = 0.8. Effector and reporter strains were mixed at a ratio of 9:1 (v/v) and infiltrated into fully expanded leaves of 4‐week‐old *N. benthamiana* plants using a 1 mL syringe. After 24 h darkness at 24°C, plants were transferred to normal growth conditions (16 h light/8 h dark) for an additional 48 h. The fluorescence signals of the leaves were observed using Lumazone SOPHIA 2048B (Princeton). The primers used are listed in Table S3.

### 4.13 Data Analysis

Data are presented as means ± standard deviation (SD) from at least three independent biological replicates. Statistical comparisons were performed with IBM SPSS Statistics v19.0 (IBM Corp, Armonk, NY). The Student's *t*‐test (*p < 0.05; **p < 0.01; ***p < 0.001; ****p < 0.0001) and one‐way ANOVA (*p* < 0.05) were used for statistical analysis.

## Author Contributions

J.X., Z.H., W.W. and P.L. conceived and designed the research. Y.L., L.Y., M.C., Y.S., J.L., Y.Z., K.L. and Y.W. performed the experiments. Y.H., W.X. and H.H. generated the banana transgenic lines. J.F., W.L., P.L. and W.W. analysed the data. Y.L., P.L., W.W., Z.H. and J.X. wrote the manuscript.

## Funding

This work was supported by the State Key Laboratory of Tropical Crop Breeding (No. NKLTCBCXTD27, NKLTCBCXTD26), the Central Public‐interest Scientific Institution Basal Research Fund (1630052022006, CATASCXTD202309), the National Natural Science Foundation of China (U22A20487) and the China Agriculture Research System (CARS‐31).

## Conflicts of Interest

The authors declare no conflicts of interest.

## Supporting information


**Figure S1:** MaERF110 localises to the nucleus.
**Figure S2:** MaERF110 negatively regulates the lignin content in Arabidopsis.

**Table S1:** Lignin content of banana accessions.
**Table S2:** List of the total genes located in 100 Kb windows of associated SNP using BLUP data.
**Table S3:** Primer sequences used in this study.

**Data S1:** Phylogenetic analysis of AP2/ERF proteins.
**Data S2:** AP2/ERF protein sequences used for phylogenetic tree construction.
**Data S3:** Phylogenetic analysis of MYB proteins.
**Data S4:** MYB protein sequences used for phylogenetic tree construction.

## Data Availability

The data that support the findings of this study are openly available in NCBI at https://www.ncbi.nlm.nih.gov/, reference number PRJNA1331702.
