# Supplementary material for: A MaERF110‐MaMYB308 Transcriptional Module Negatively Regulates Lignin‐Mediated Defence Against Fusarium Wilt in Banana
Source: Plant Biotechnol J. 2026 Jan 6;24(5):2811–25. doi: 10.1111/pbi.70528 (PMC13110180; doi:10.1111/pbi.70528)
Supplement: Supplementary file 1 — Figure S1: MaERF110 localises to the nucleus. Figure S2: MaERF110 negatively regulates the lignin content in Arabidopsis. [file PBI-24-2811-s003.docx]

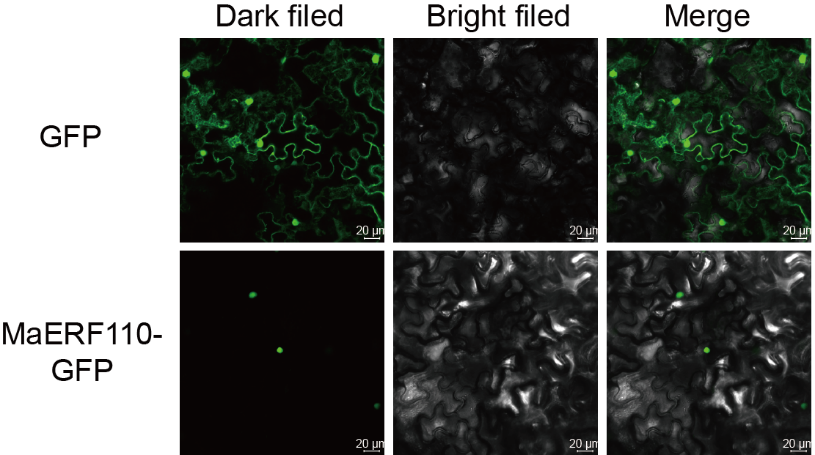


**Figure S1. MaERF110 localizes to the nucleus.** Confocal images of *Nicotiana benthamiana* leaf epidermal cells transiently expressing *35S::MaERF110-GFP*. Green fluorescence (green) is restricted to nuclei, confirming the nuclear localization of MaERF110. Scale bars = 20 μm.


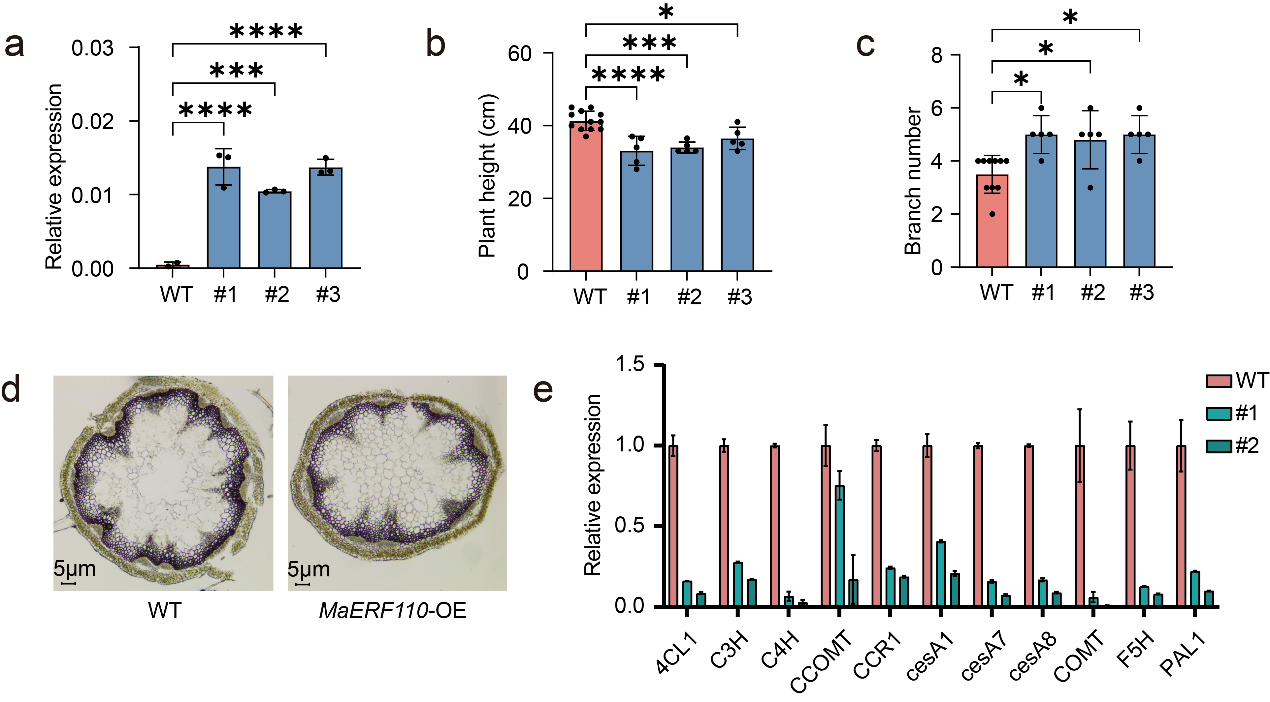


**Figure S2. *MaERF110* negatively regulates the lignin content in *Arabidopsis.***

**(a)** Relative *MaERF110* transcript levels in WT and two independent OE lines. Data are means ± SD (n = 3). **(b, c)** Plant height (**b**) and branch number (**c**) of *MaERF110*-OE lines and WT *Arabidopsis*. Data are means ± SD (n ≥ 5). **(d)** Phloroglucinol-HCl staining of basal stem cross-sections; lignified xylem is markedly reduced in OE lines. Scale bars = 5 μm. **(e)** Expression of core lignin pathway genes *4CL1*, *C3H*, *C4H*, *CCOMT*, *CCR1*, *cesA*, *COMT*, *F5H*, *PAL1* in *MaERF110*-OE lines and WT *Arabidopsis*. Data are means ± SD (n = 3). Asterisks represent significant differences (**p* < 0.05; ***p* < 0.01; ****p* < 0.001; *****p* < 0.0001).
