# Supplementary material for: A MaERF110‐MaMYB308 Transcriptional Module Negatively Regulates Lignin‐Mediated Defence Against Fusarium Wilt in Banana
Source: Plant Biotechnol J. 2026 Jan 6;24(5):2811–25. doi: 10.1111/pbi.70528 (PMC13110180; doi:10.1111/pbi.70528)
Supplement: Supplementary file 3 — Data S1: Phylogenetic analysis of AP2/ERF proteins. Data S2: AP2/ERF protein sequences used for phylogenetic tree construction. Data S3: Phylogenetic analysis of MYB proteins. Data S4: MYB protein sequences used for phylogenetic tree construction. [file PBI-24-2811-s002.docx]

**Supplementary File 1. Phylogenetic analysis of AP2/ERF proteins.**

(((((OsERF98:0.02511948,OsERF114:0.06831452)1.0000:0.22077676,LOC_Os04g34970:0.30269765)0.9940:0.12169265,(OsEREBP2:0.34436807,OsERF105:0.39821257)0.9780:0.08689930)0.8870:0.05424048,(OsERF110:0.51129481,(MaERF110:0.47136567,AtRAP2.6:0.28702203)0.8840:0.08443378)0.3030:0.00979289)0.6010:0.04764417,(AtRAP2.6L:0.31692587,(AtERF115:0.16534469,AtERF114:0.14831286)1.0000:0.24539810)0.9990:0.16983409,(AtERF112:0.53474237,(AtERF110:0.24617305,AtERF111:0.52248169)0.9200:0.08448799)0.5110:0.02501847);

**Supplementary File 2. AP2/ERF Protein sequences used for phylogenetic tree construction.**

>MaERF110

MCFKVASDTGAGDGGGGSDGEAHVPPTGRLTSSFMEYERSVMVSALVHVVAGGRGTEAPPGGGNGQTRWNEGFVHEAASELHASFGDFGRSSSSAASADATEQTAAPAAEAAAAMAGTEQGERVRYRGVRRRPWGKWAAEIRDPHKAARVWLGTFDTAEAAARAYDEAALRFRGNRAKLNFPEEAGLRKAPAVTPEAHMESQPFGRHGASGSTAAVAARDYMEYSRLLRGEGEYQRMPPTALLDQMMYSGASAASPFTDASLASSSFASAPFPPSSPLIYPPESEQQMDYLQPPPWSGCSHYPPSSSSH

>AtRAP2.6

MVSMLTNVVSGETEPSASATWTMGHKREREEFSLPPQPLITGSAVTKECESSMSLERPKKYRGVRQRPWGKWAAEIRDPHKATRVWLGTFETAEAAARAYDAAALRFRGSKAKLNFPENVGTQTIQRNSHFLQNSMQPSLTYIDQCPTLLSYSRCMEQQQPLVGMLQPTEEENHFFEKPWTEYDQYNYSSFG

>AtERF112

MHSGKRPLSPESMAGNREEKKELCCCSTLSESDVSDFVSELTGQPIPSSIDDQSSSLTLQEKSNSRQRNYRGVRQRPWGKWAAEIRDPNKAARVWLGTFDTAEEAALAYDKAAFEFRGHKAKLNFPEHIRVNPTQLYPSPATSHDRIIVTPPSPPPPIAPDILLDQYGHFQSRSSDSSANLSMNMLSSSSSSLNHQGLRPNLEDGENVKNISIHKRRK

>AtERF115

MANSGNYGKRPFRGDESDEKKEADDDENIFPFFSARSQYDMRAMVSALTQVIGNQSSSHDNNQHQPVVYNQQDPNPPAPPTQDQGLLRKRHYRGVRQRPWGKWAAEIRDPQKAARVWLGTFETAEAAALAYDNAALKFKGSKAKLNFPERAQLASNTSTTTGPPNYYSSNNQIYYSNPQTNPQTIPYFNQYYYNQYLHQGGNSNDALSYSLAGGETGGSMYNHQTLSTTNSSSSGGSSRQQDDEQDYARYLRFGDSSPPNSGF

>AtRAP2.6L

MVSALSRVIENPTDPPVKQELDKSDQHQPDQDQPRRRHYRGVRQRPWGKWAAEIRDPKKAARVWLGTFETAEEAALAYDRAALKFKGTKAKLNFPERVQGPTTTTTISHAPRGVSESMNSPPPRPGPPSTTTTSWPMTYNQDILQYAQLLTSNNEVDLSYYTSTLFSQPFSTPSSSSSSSQQTQQQQLQQQQQQREEEEKNYGYNYYNYPRE

>AtERF110

MSAMVSALTQVVSARSQTEAEGAHSSSSSAGHKRGWLGIDSAPIPSSFARVDSSHNPIEESMSKAFPEEAREKKRRYRGVRQRPWGKWAAEIRDPHRAARVWLGTFDTAEAAARAYDEAALRFRGNKAKLNFPEDVRILPPPPPLLRSPADTVANKAEEDLINYWSYTKLLQSSGQRSFLERGQEESSNIFEHSPMEQPLPPSSSGPSSSNFPAPSLPNT

>AtERF114

MYGKRPFGGDESEEREEDENLFPVFSARSQHDMRVMVSALTQVIGNQQSKSHDNISSIDDNYPSVYNPQDPNQQVAPTHQDQGDLRRRHYRGVRQRPWGKWAAEIRDPKKAARVWLGTFETAESAALAYDEAALKFKGSKAKLNFPERVQLGSNSTYYSSNQIPQMEPQSIPNYNQYYHDASSGDMLSFNLGGGYGSGTGYSMSHDNSTTTAATTSSSSGGSSRQQEEQDYARFWRFGDSSSSPHSGY

>AtERF111

MCVLKVANQEDNVGKKAESIRDDDHRTLSEIDQWLYLFAAEDDHHRHSFPTQQPPPSSSSSSLISGFSREMEMSAIVSALTHVVAGNVPQHQQGGGEGSGEGTSNSSSSSGQKRRREVEEGGAKAVKAANTLTVDQYFSGGSSTSKVREASSNMSGPGPTYEYTTTATASSETSSFSGDQPRRRYRGVRQRPWGKWAAEIRDPFKAARVWLGTFDNAESAARAYDEAALRFRGNKAKLNFPENVKLVRPASTEAQPVHQTAAQRPTQSRNSGSTTTLLPIRPASNQSVHSQPLMQSYNLSYSEMARQQQQFQQHHQQSLDLYDQMSFPLRFGHTGGSMMQSTSSSSSHSRPLFSPAAVQPPPESASETGYLQDIQWPSDKTSNNYNNSPSS

>OsEREBP2

MTVAGASELMSGYYQAQEMSTMVSALARVVAGGGGGGGDGDGDQWAWSSPSPSSSAAAAAARGVQERRREEQAMHELAGYACGGAPSPEFAGSEQSSDTQSASAATMDEHHSPVGGGGNAEGPDTPRRRYRGVRQRPWGKWAAEIRDPHKAARVWLGTFETAEAAARAYDEAALRFRGSRAKLNFPEDARLYPTAATTTTAAPPPAPVAAASPSAAIYPGASQSAEYLRYQMLLQGRLTTATPNQGTLLPFYGGGGGGGSMTNPYGGGGGGAMSGFLGSYYSFPTPSVSVATVPSSTSSAPGNYYSSHGGSHQSMSAAEEWNWENALVYPATAASWSESSYHHHPPPPHTQ*

>OsERF98

MAALFEAAETAAIVAALTRVIADGGRGGGGGVCVPPPAPSLVVPPRAGTGGGRRVDVAREEEMVGVVSAGDHAGGANVAEAAAAVVVAAPATARRYRGVRRRPWGKWAAEIRDPRKAARVWLGTFRTAEDAARAYDAAALRFRGRRAKLNFPEEVSRPWQGHDVDHMSCSPPSISNARFLGSWTFGPPQPPSRSVAAAATTLLGGSHGGNGAENGRE*

>OsERF114

MAALFEAAETAAIVAALTRVIADGGRGGSGAGVPPPAPSLVVPPLAGTGGGRRVDVAREEEMVGVVSAGDHTGEASVAAAGVVVAAPATARRYRGVRRRPWGKWAAEIRDPRKAARVWLGTFRTTEDAARAYDAAVLRFRGRRAKLNFPEEASRPRRPWKGHDVDHMSCSPPSIANARFLGSWIFGPPPPSRSVAAATTTLLGGSHGSNGADNGRE*

>LOC_Os04g34970

MALSEPDLAAETEAIVSALTHVVAYGGGGPPPSEETAASAVTRTAPWRADGARQGAVPAARKYRGVRRRPWGKWAAEIRDPHRAARVWLGTFATAEDAARAYDAAALRFRGGRARLNFPEDAAAADERRATDAAAAAAAASSAPAALLESQPGDVADCLDYSRILAGAPERPTSSTVPDGFFGGGGNGRFLHSWSIGTSPSPSGSGSGGAGGGGGGGGGGAPVRPLFHGGNGWEQRGDSA

YNGF*

>OsERF105

MADQRRRFRGGGDWQASVDDVVDDGGELEAAAAAAARGSVLSGEYQAQEMSTMVSALTWVVAAGHDDHGGGQWSGLVDVPATTLAGGGGGDYGHGAQGSYYYYGAAPTSTPEFVAGGQQEQLSSDVPQGGASLGLAMDEHSPTYTVEASSSADQHGGGGGGRRYRGVRQRPWGKWAAEIRDPHKAARVWLGTFETAEAAARAYDEAALRFRGSRAKLNFPEDARLSSPPAGAGAGGATAAAQTVPVAYPASAVSDYLQYQMLLHGGGGGGGGRYPLYYGGGAAAAMSSSLGPYSSIPTSSVTVASVPSSSSAASSSSGYGAPAEHGEAVQWTSWPDGGGWTYPATTSSWSGSSQYPPPPRPPQQ*

>OsERF110

MTIFKMNHNISLISFTIAFSTLFRLDFAEHAAAMAAGQYAPATSSAMASPREQASSPSSGDAAGGGGGGGRKRYRGVRQRPWGKWAAEIRDPVKAARVWLGTFDTAEAAARAYDDAALRFRGCRAKLNFPEDAALLPPPPPPPAPAPAPPQSQGMVGVGEEYSEYARFLQGAGEPPHFLEQIMEDSPRPSTAAGASSSSSGQSSFPLFYSFAGHELGGNEANLARPPESGGAGGDGGRGSSPPVTWPGYGWGAPPPWDPSR*

**Supplementary File 3. Phylogenetic analysis of MYB proteins.**

((((((AtMYB7:0.14604701,AtMYB32:0.15850762)0.9990:0.08441473,AtMYB4:0.23686115)0.9700:0.04334023,MaMYB306:0.19525909)0.8970:0.03938170,AtMYB3:0.29836031)0.7770:0.03483982,(AtMYB8:0.15103634,AtMYB6:0.15569392)0.9730:0.07588580)0.9930:0.11319996,AtMYB111:0.46431122,(AtMYB12:0.19613060,AtMYB11:0.23418051)1.0000:0.30337863);

**Supplementary File 4. MYB protein sequences used for phylogenetic tree construction.**

>MaMYB306

MGRSPCCEKAHTNKGAWTKEEDEKLISYIKAHGEGCWRSLPKAAGLLRCGKSCRLRWINYLRPDLKRGNFTAEEDDLIIKLHGLLGNKWSLIAGRLPGRTDNEIKNYWNTHIKRKLLSRGLDPKTHRPIDVDRPFAALKQRDMSAAQDSTASEKYFSGDEEGGSGSTGRHKGCYLDLNLDLSISLPYHHPCQEQSSPREAKSQATPRSLRCHHQWNL

>AtMYB3

MGRSPCCEKAHMNKGAWTKEEDQLLVDYIRKHGEGCWRSLPRAAGLQRCGKSCRLRWMNYLRPDLKRGNFTEEEDELIIKLHSLLGNKWSLIAGRLPGRTDNEIKNYWNTHIKRKLLSRGIDPNSHRLINESVVSPSSLQNDVVETIHLDFSGPVKPEPVREEIGMVNNCESSGTTSEKDYGNEEDWVLNLELSVGPSYRYESTRKVSVVDSAESTRRWGSELFGAHESDAVCLCCRIGLFRNESCRNCRVSDVRTH

>AtMYB8

MGRSPCCEKAHMNKGAWTKEEDQRLIDYIRNHGEGSWRSLPKSVGLLRCGKSCRLRWINYLRPDLKRGNFTDGEEQIIVKLHSLFGNKWSLIAGKLPGRTDNEIKNYWNTHIKRKLLNRGIDPKTHGSIIEPKTTSFHPRNEDLKSTFPGSVKLKMETSCENCASTSGTTTDEDLRLSVDCDYRYDHLDKELNLDLTLGYSPTRFVGVGSCY

>AtMYB7

MGRSPCCEKEHMNKGAWTKEEDERLVSYIKSHGEGCWRSLPRAAGLLRCGKSCRLRWINYLRPDLKRGNFTHDEDELIIKLHSLLGNKWSLIAARLPGRTDNEIKNYWNTHIKRKLLSKGIDPATHRGINEAKISDLKKTKDQIVKDVSFVTKFEETDKSGDQKQNKYIRNGLVCKEERVVVEEKIGPDLNLELRISPPWQNQREISTCTASRFYMENDMECSSETVKCQTENSSSISYSSIDISSSNVGYDFLGLKTRILDFRSLEMK

>AtMYB12

MGRAPCCEKVGIKRGRWTAEEDQILSNYIQSNGEGSWRSLPKNAGLKRCGKSCRLRWINYLRSDLKRGNITPEEEELVVKLHSTLGNRWSLIAGHLPGRTDNEIKNYWNSHLSRKLHNFIRKPSISQDVSAVIMTNASSAPPPPQAKRRLGRTSRSAMKPKIHRTKTRKTKKTSAPPEPNADVAGADKEALMVESSGAEAELGRPCDYYGDDCNKNLMSINGDNGVLTFDDDIIDLLLDESDPGHLYTNTTCGGDGELHNIRDSEGARGFSDTWNQGNLDCLLQSCPSVESFLNYDHQVNDASTDEFIDWDCVWQEGSDNNLWHEKENPDSMVSWLLDGDDEATIGNSNCENFGEPLDHDDESALVAWLLS

>AtMYB11

MGRAPCCEKVGIKKGRWTAEEDRTLSDYIQSNGEGSWRSLPKNAGLKRCGKSCRLRWINYLRSDIKRGNITPEEEDVIVKLHSTLGTRWSTIASNLPGRTDNEIKNYWNSHLSRKLHGYFRKPTVANTVENAPPPPKRRPGRTSRSAMKPKFILNPKNHKTPNSFKANKSDIVLPTTTIENGEGDKEDALMVLSSSSLSGAEEPGLGPCGYGDDGDCNPSINGDDGALCLNDDIFDSCFLLDDSHAVHVSSCESNNVKNSEPYGGMSVGHKNIETMADDFVDWDFVWREGQTLWDEKEDLDSVLSRLLDGEEMESEIRQRDSNDFGEPLDIDEENKMAAWLLS

>AtMYB6

MGRSPCCEKAHTNKGAWTKEEDQRLVDYIRNHGEGCWRSLPKSAGLLRCGKSCRLRWINYLRPDLKRGNFTDDEDQIIIKLHSLLGNKWSLIAGRLPGRTDNEIKNYWNTHIKRKLLSHGIDPQTHRQINESKTVSSQVVVPIQNDAVEYSFSNLAVKPKTENSSDNGASTSGTTTDEDLRQNGECYYSDNSGHIKLNLDLTLGFGSWSGRIVGVGSSADSKPWCDPVMEARLSLL

>AtMYB32

MGRSPCCEKDHTNKGAWTKEEDDKLISYIKAHGEGCWRSLPRSAGLQRCGKSCRLRWINYLRPDLKRGNFTLEEDDLIIKLHSLLGNKWSLIATRLPGRTDNEIKNYWNTHVKRKLLRKGIDPATHRPINETKTSQDSSDSSKTEDPLVKILSFGPQLEKIANFGDERIQKRVEYSVVEERCLDLNLELRISPPWQDKLHDERNLRFGRVKYRCSACRFGFGNGKECSCNNVKCQTEDSSSSSYSSTDISSSIGYDFLGLNNTRVLDFSTLEMK

>AtMYB4

MGRSPCCEKAHTNKGAWTKEEDERLVAYIKAHGEGCWRSLPKAAGLLRCGKSCRLRWINYLRPDLKRGNFTEEEDELIIKLHSLLGNKWSLIAGRLPGRTDNEIKNYWNTHIRRKLINRGIDPTSHRPIQESSASQDSKPTQLEPVTSNTINISFTSAPKVETFHESISFPGKSEKISMLTFKEEKDECPVQEKFPDLNLELRISLPDDVDRLQGHGKSTTPRCFKCSLGMINGMECRCGRMRCDVVGGSSKGSDMSNGFDFLGLAKKETTSLLGFRSLEMK

>AtMYB111

MGRAPCCEKIGLKRGRWTAEEDEILTKYIQTNGEGSWRSLPKKAGLLRCGKSCRLRWINYLRRDLKRGNITSDEEEIIVKLHSLLGNRWSLIATHLPGRTDNEIKNYWNSHLSRKIYAFTAVSGDGHNLLVNDVVLKKSCSSSSGAKNNNKTKKKKKGRTSRSSMKKHKQMVTASQCFSQPKELESDFSEGGQNGNFEGESLGPYEWLDGELERLLSSCVWECTSEEAVIGVNDEKVCESGDNSSCCVNLFEEEQGSETKIGHVGITEVDHDMTVEREREGSFLSSNSNENNDKDWWVGLCNSSEVGFGVDEELLDWEFQGNVTCQSDDLWDLSDIGEITLE
